# Supplementary material for: 3D (x-y-t) Raman imaging of tomato fruit cuticle: Microchemistry during development
Source: Plant Physiol. 2022 Aug 16;191(1):219–32. doi: 10.1093/plphys/kiac369 (PMC9806558; doi:10.1093/plphys/kiac369)
Supplement: kiac369_Supplementary_Data [file kiac369_supplementary_data.pdf]

Supplemental Data

Supplemental Table S1. Raman band assignment of chalconaringenin in gas phase according to DFT calculations.

Theoretical ṽ (cm^{-1}) in gas phase	Experimental ṽ (cm^{-1}) in chalconaringenin spectrum	Experimental ṽ (cm^{-1}) in cuticle Raman spectrum	Assignment	Eigenvectors
544	546	545	C=C stretching ring B + C=C bending ring A and vinyl group	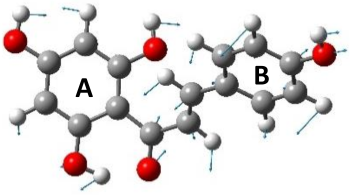
1489	1509	-	C=O stretching + O-H bending (H bond) + C=C stretching of ring A + C=C bending of ring B	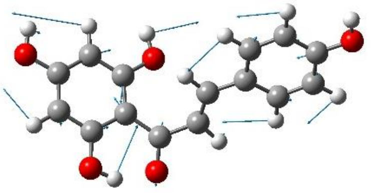

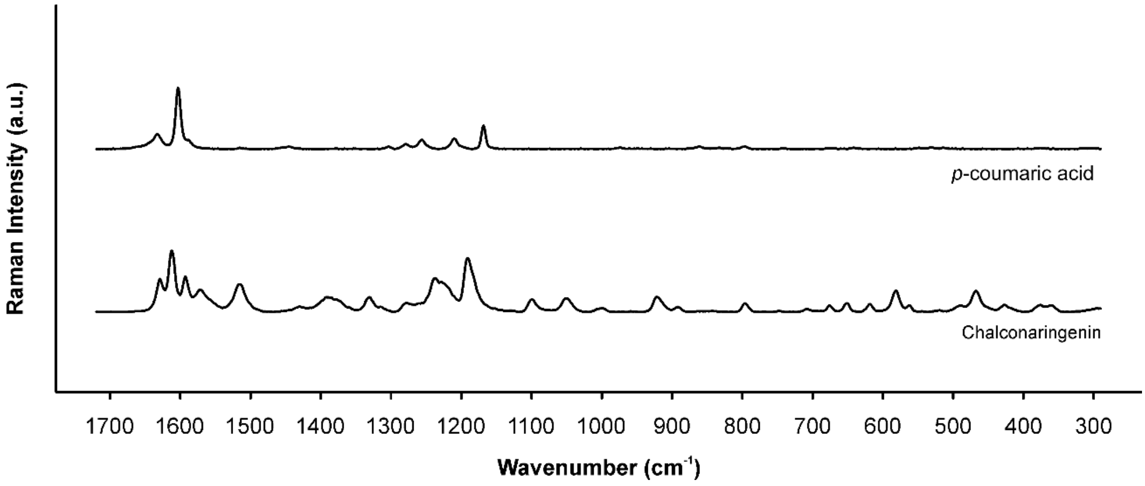

Supplemental Figure S1. Raman spectra of the free conformations of *p*-coumaric acid and chalconaringenin.

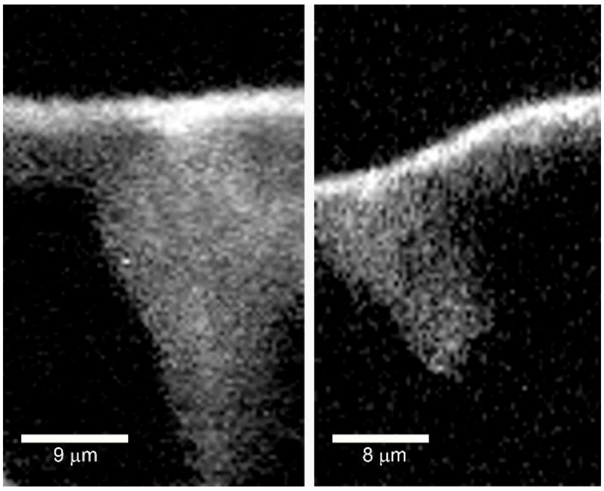

Supplemental Figure S2. Raman images corresponding to a univariate data analysis of phenolic acids in tomato cuticle cross-sections before (left) and after (right) epicuticular wax removal. Raman images of 'Cascada' fruit cuticles at 15 daa (days after anthesis) before (left) and after (right) extraction of epicuticular waxes**.** These images have been generated for the integration of the 1622-1642 cm^{-1} band mainly attributed to phenolic acids. Images were scaled from minimum (black) to maximum (bright colors).

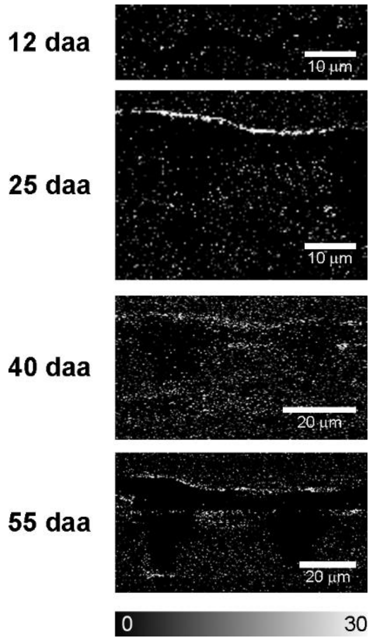

Supplemental Figure S3. Raman images based on integration of a band related to the presence of a superficial waxy layer. Raman images were generated by integrating over 1127-1137 cm^{-1} (waxes) of 'Cascada' fruit cuticles from different stages of fruit development. The grey scale of the Raman images a represents CCDcts (Charge Coupled Device counts).

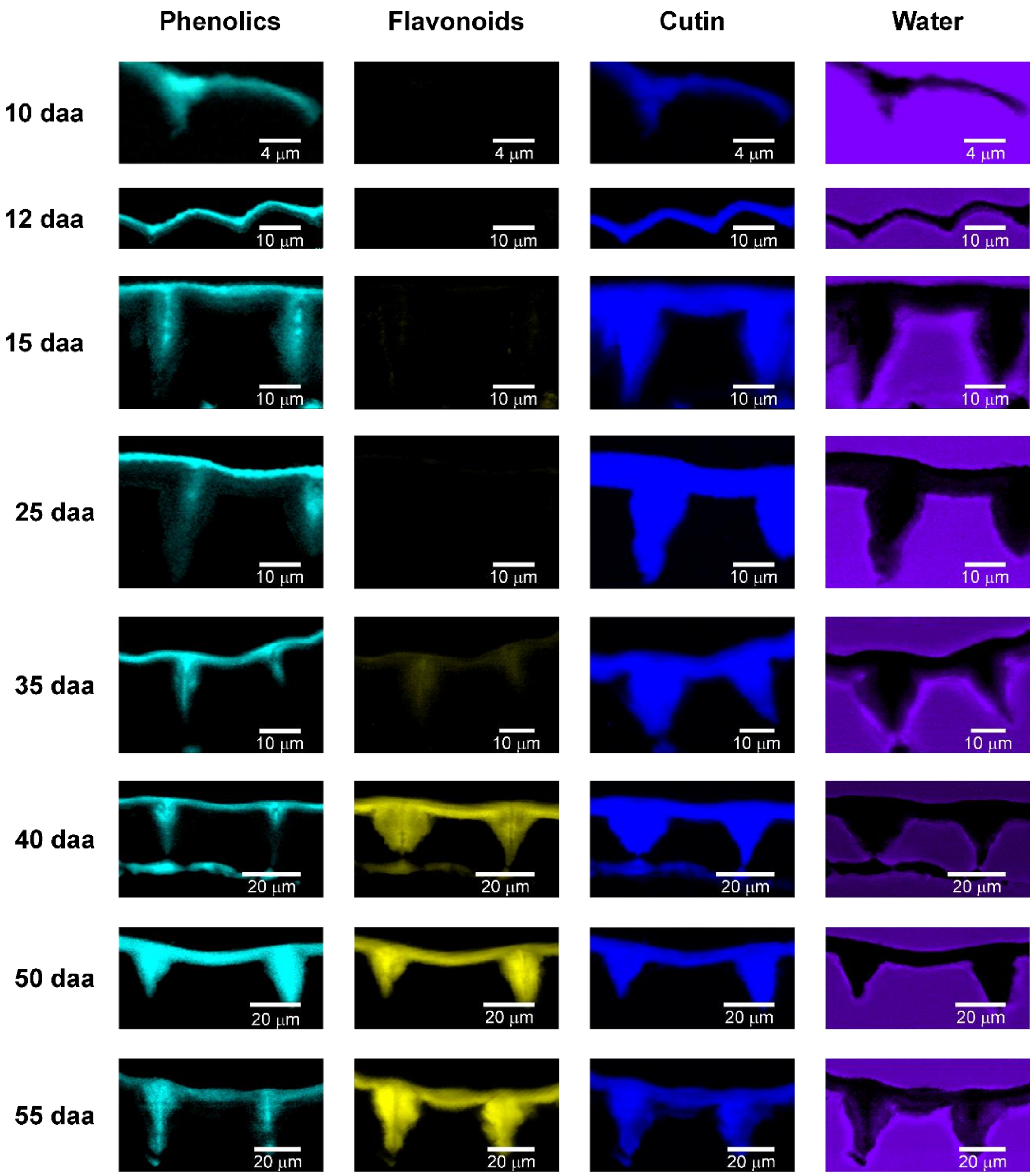

Supplemental Figure S4. Basis analysis using the purest spectra obtained from the NMF algorithm. Distribution maps after a basis analysis of different cuticle components in cross-sections of 'Cascada' fruit cuticles throughout development, from 10 to 55 daa (days after anthesis). Location of phenolics acids, flavonoids with a contribution of phenolic acids, the cutin (cutin + polysaccharides + waxes) and water are shown in turquoise, yellow, blue and purple, respectively. False colors within each image were scaled to minimum (black) to maximum (bright colors).

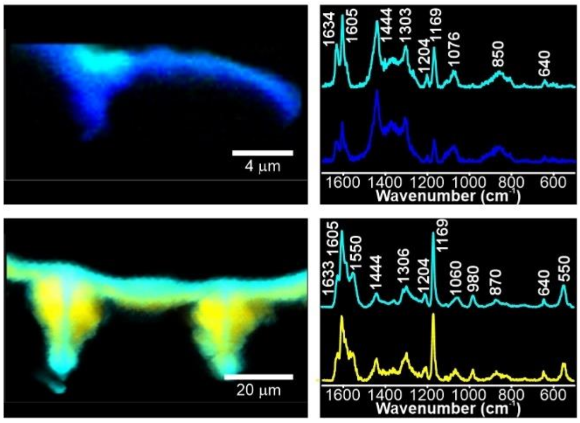

Supplemental Figure S5. Distribution maps and Raman spectra obtained by NMF at 10 daa and 55 daa. Abundance maps of ‘Cascada’ fruit cuticles at 10 and 55 daa. They are accompanied by extracted spectra of each basis as obtained by NMF. Turquoise, blue and yellow colors represent the basis with dominant contribution of phenolics, cutin and flavonoids, respectively. A black canvas was added to the cross-sections for aesthetic reasons. daa, days after anthesis

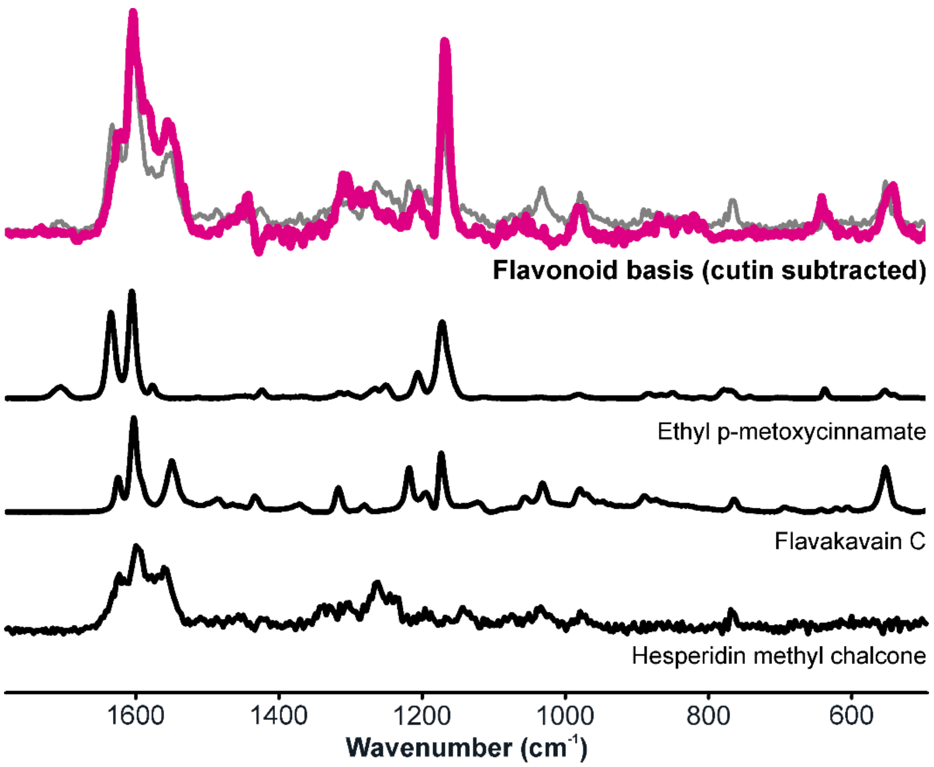

Supplemental Figure S6. Mixture analysis of the basis spectra of the flavonoid spectra after subtracting the contribution of cutin (pink spectrum). The basis spectrum fit solely with phenolics and flavonoids from the library. Model fit spectra based on OMP are shown in grey.

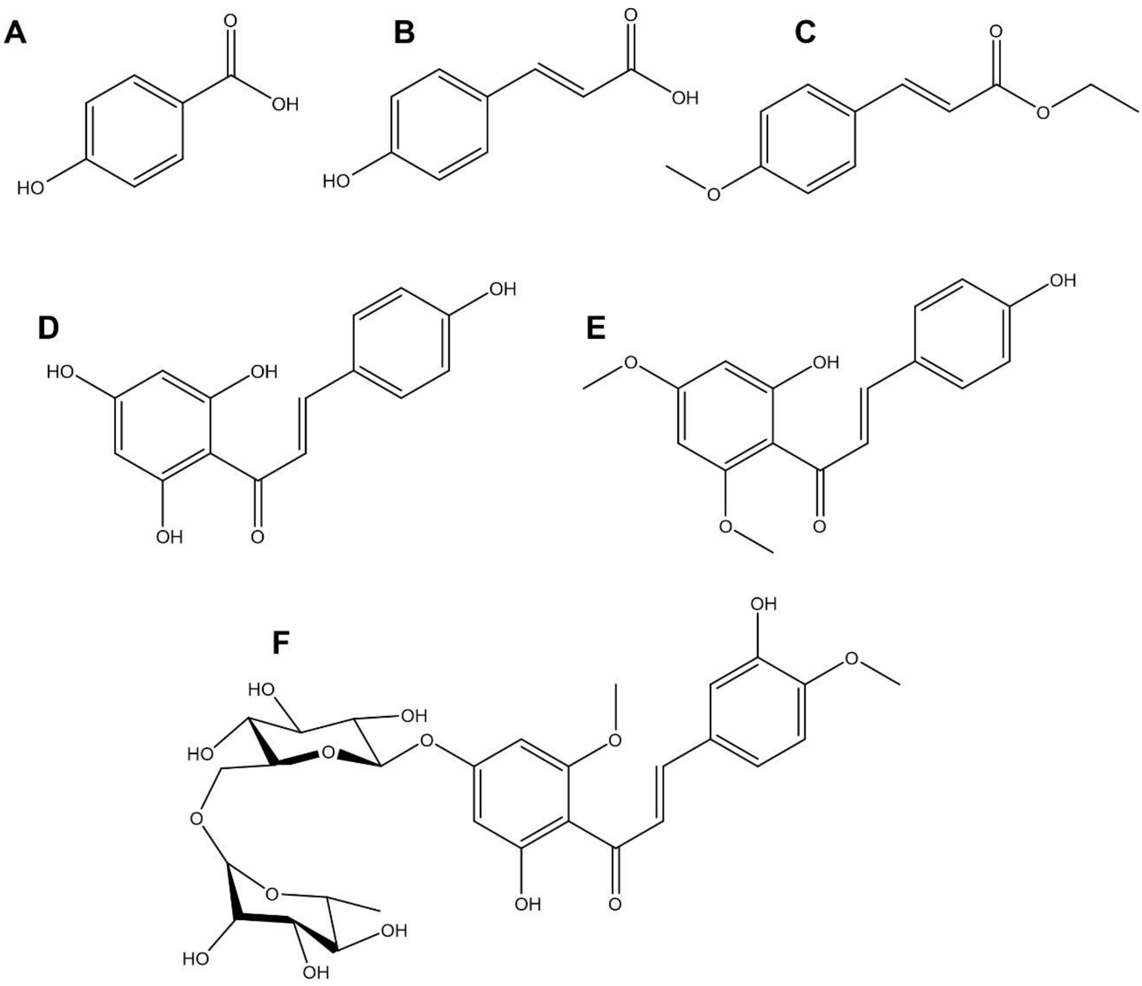

Supplemental Figure S7. Chemical structure of phenolic compounds mentioned in this work. Chemical structure of **(A)** *p*-hydroxybenzoic acid **(B)** *p*-coumaric acid **(C)** ethyl *p*-methoxycinnamate **(D)** chalconaringenin **(E)** flavokavain C **(F)** hesperidin methyl chalcone.
